# Revealing determinants of two‐phase dynamics of P53 network under gamma irradiation based on a reduced 2D relaxation oscillator model

**DOI:** 10.1049/iet-syb.2017.0041

**Published:** 2018-02-01

**Authors:** Gökhan Demirkıran, Güleser Kalaycı Demir, Cüneyt Güzeliş

**Affiliations:** ^1^ Department of Electrical‐Electronics Engineering Yaşar University Bornova İzmir 35100 Turkey; ^2^ The Graduate School of Natural and Applied Sciences Dokuz Eylül University Buca İzmir 35160 Turkey; ^3^ Department of Electrical and Electronics Engineering Dokuz Eylül University Buca İzmir 35160 Turkey

**Keywords:** physiological models, cellular biophysics, cancer, difference equations, delays, enzymes, biochemistry, molecular biophysics, gamma‐rays, radiation therapy, two‐phase dynamics model, P53 network, gamma irradiation, 2D relaxation oscillator model, ATM model, Wip1 variables, p53‐regulators, cell fate decision, excitable relaxation oscillator, Wip1 time delay, state‐dependent delay differential equation, cell cycle arrest, cell apoptosis, cancer therapies, Wip1 overexpression, Wip1 downregulation, ATM deficiency, Mdm2 overexpression, Mdm2 downregulation, mutation effects, phase space approach

## Abstract

This study proposes a two‐dimensional (2D) oscillator model of p53 network, which is derived via reducing the multidimensional two‐phase dynamics model into a model of ataxia telangiectasia mutated (ATM) and Wip1 variables, and studies the impact of p53‐regulators on cell fate decision. First, the authors identify a 6D core oscillator module, then reduce this module into a 2D oscillator model while preserving the qualitative behaviours. The introduced 2D model is shown to be an excitable relaxation oscillator. This oscillator provides a mechanism that leads diverse modes underpinning cell fate, each corresponding to a cell state. To investigate the effects of p53 inhibitors and the intrinsic time delay of Wip1 on the characteristics of oscillations, they introduce also a delay differential equation version of the 2D oscillator. They observe that the suppression of p53 inhibitors decreases the amplitudes of p53 oscillation, though the suppression increases the sustained level of p53. They identify Wip1 and P53DINP1 as possible targets for cancer therapies considering their impact on the oscillator, supported by biological findings. They model some mutations as critical changes of the phase space characteristics. Possible cancer therapeutic strategies are then proposed for preventing these mutations’ effects using the phase space approach.

## 1 Introduction

Cell responds to some particular stresses via tumour suppressor p53 network. p53 network determines the cell fate when confronted with these stresses, depending on the stress type and severity [1]. Among these stresses, the most deleterious one is gamma irradiation, which causes double strand breaks (DSBs) in DNA. In [2, 3], experimental studies are done to investigate how gamma irradiation modulates p53 (the product of p53 gene) dynamics and how it affects cell fate outcomes. They observed that p53 (concentration) level oscillates under gamma irradiation and reported that the peak amplitude and period did not depend on the dose of irradiation but the number of pulses correlates with the level of DNA damage. Lahav *et al.* [2] termed this type of oscillation as digital pulses. After the publication of the results of these experiments, there have been various approaches to model this oscillatory behaviour occurring under gamma irradiation [4].

The question of how p53 dynamics determines the cell fate also attracts the attention of many researchers. In a wet‐lab experiment, Purvis *et al.* [5] showed that p53 pulses and sustained (stable steady state) p53 level lead to activation of two different sets of downstream genes related to cell fate decisions, which means p53 dynamics itself directly influences cell fate. Zhang *et al.* [6] proposed a mathematical model to describe these observations. Their model has the following features. Different phosphorylation forms of p53 (p53arrester and p53killer) are utilised to give different cell fate outcomes. DSB complexes (DSBCs) stimuli due to gamma irradiation result in repeated p53 pulses. If the damage is not repaired in a certain amount of time, i.e. damage is too severe, repeated pulses are replaced by sustained p53 level initiating apoptosis through caspase mechanism. Zhang's model successfully shows digital pulses and sustained p53 level in two distinct phases, namely the transient oscillation of p53 and the succeeding stable equilibrium of a high‐level p53. We focus on this two‐phase dynamics model of p53 network, and model it as a two‐dimensional (2D) oscillator by reducing the whole multi‐dimensional model. We show that the introduced oscillator is of relaxation type that is abundantly found in biological systems. Our 2D relaxation oscillator model of p53 dynamics shows diverse behaviours underpinning cell fate such that its mode determines the physiological states of the cell. Since p53 dynamics has a direct influence on cellular fate decision [5], developing of a 2D oscillator model capable of describing p53 dynamics is of importance in several aspects including the introduction of novel cancer therapeutic strategies. The analysis on the proposed 2D oscillator model explains a part of the known phenomena seen in p53 network, reveals a set of determinants of p53 network, provides predictions for p53 dynamics to be validated and confirms some biological findings as stated below:

(i) We show via in‐silico studies that Mdm2_n_ (nuclear Mdm2), known as the main negative regulator of p53, has different effects on the first and second phases of the p53 dynamics. It is observed that decreasing of [Mdm2_n_], i.e. the concentration of Mdm2_n_ protein, results unexpectedly in a smaller amplitude of p53 oscillation yielding a weaker cell cycle arrest signal in the first phase, whereas it causes, as expected, an increase in the steady‐state level of p53, so producing a positive effect on apoptosis in the second phase.
(ii) We identify that decreasing p53 inhibitors, e.g. Mdm2, as a cancer therapeutic approach to activate p53 function, may have a serious side effect: it may result in weak oscillations of p53 that causes problem in arresting cell cycle.
(iii) We found that the oscillations in p53 network is due to an underlying oscillator that is of relaxation type, which means the p53 network has more complex dynamics than a simple static feedforward model of suppressor–effector interaction.
(iv) We detected that Wip1 (wild‐type p53‐induced phosphatase 1, the product of PP1MD gene) and P53DINP1 (p53‐dependent damage inducible nuclear protein 1) regulators have profound effects on the cell fate due to their certain roles in the oscillator. Wip1 dynamics is observed to have a strong effect on the frequency and amplitude of oscillations and P53DINP1 is understood to be an oscillation accumulation triggered genetic switch (OATGS), which shuts off Wip1 feedback loop to provide sustained level of p53* (active p53 protein) to drive the cell to apoptosis. These findings may pave the way for some alternative approaches of developing new therapeutic drugs.
(v) We show that mutations such as Wip1 overexpression and ATM deficiency drastically change the phase space of the reduced 2D oscillator model of p53 network and result in malfunctioning of the oscillator. We show mathematically that the oscillatory phase space can be recovered and so apoptosis can be initiated in the types of cancer cells caused by Wip1 overexpression or ATM deficiency as with suppression of Wip1 overexpression and degradation of Wip1. So, it is observed that such a phase space analysis of Wip1 and ATM dynamics provides a tool to manipulate the cancer cells pharmacologically.
(vi) The presented work contributes to the systems level understanding of p53 network, so providing a better interpretation of wet‐lab experiments and suggests specific targets, namely Wip1 and P53DINP1, to be investigated with further drug researches.

This paper is organised as follows. In Section 2, we review the two‐phase dynamics model of Zhang *et al.* [6] and identify the core oscillator subsystem. In Section 3, we reduce the oscillator model into a 2D one and show that it is of relaxation type. We continue with the analyses on the oscillator, and describe the key roles of essential proteins from the perspective of relaxation oscillator. In Section 4, we reveal the main determinants of the p53 network oscillations. In Section 5, using the proposed oscillator model, we analyse some mutations in the phase space, and propose cancer therapeutic strategies related to these mutations based on the proposed 2D oscillator model.

## 2 Identification of 6D oscillator module in two‐phase dynamics model of Zhang *et al.* [6]

**Fig. 1:**
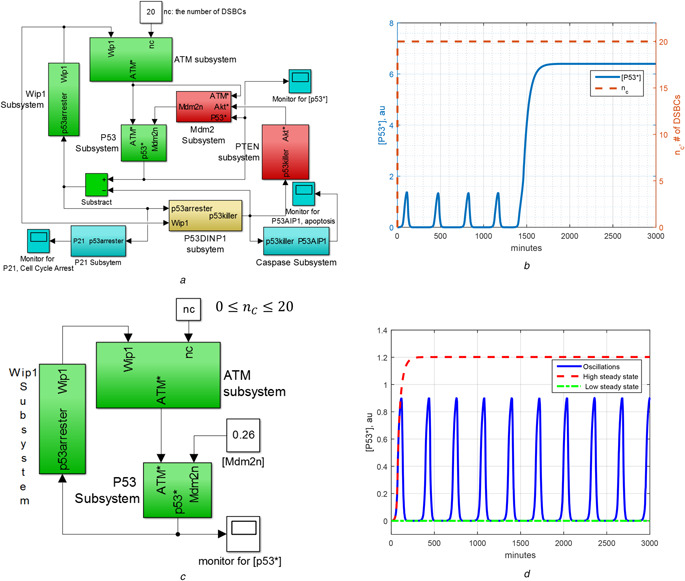
Representing two‐phase model and oscillator subsystem in MATLAB/Simulink^®^, and numerical simulations. Note that au stands for arbitrary unit ***(a)*** Block diagram representation of the two‐phase dynamics model by Zhang *et al.* [1] in MATLAB/Simulink, ***(b)*** Numerical simulation for Zhang's model: [p53*] first oscillates and, after four pulses, it goes to a higher steady‐state level initiating apoptosis, ***(c)*** Block diagram representation of the isolated oscillator subsystem of the two‐phase dynamics in MATLAB/Simulink. The variables [Mdm2_n_], [p53killer] and $n_{c}$ that are transferred from other subsystems are kept as parameters in this core oscillator subsystem, ***(d)*** Numerical results for the core oscillator subsystem defined in Table 1. Three different modes appear under the conditions provided in Table 2

Section 2 scrutinises the two‐phase dynamics of the model by Zhang *et al.* [6] as identifying the p53 network's subsystems as the main constituents responsible for the digital pulses. For this purpose, the model by Zhang *et al.*, which consists of 17 ordinary differential equations derived from enzyme kinetics, is re‐illustrated in Fig. 1*a* as a block diagram in MATLAB/Simulink^®^ environment, providing a systems theoretical framework based on the differential equation instruments.

**Table 1:** Core oscillator subsystem of the Zhang's full two‐phase dynamics model (see [1] for the definitions of model parameters)

Equation number	Equations
1	$\frac{d\left[\text{AT}M_{2}\right]}{dt}=0.5*k_{\text{dim}}[\text{ATM}]{\;}^{2}-\;k_{\text{undim}}\left[\text{AT}M_{2}\right]$
2	$\frac{d\left[\text{AT}M^{*}\right]}{dt}=k_{\text{acatm}}\frac{n_{c}}{n_{c}+j_{\text{nc}}}[\;\text{AT}M^{*}]\;\frac{[\text{ATM}]\;\;\;\;}{(\left[\text{ATM}\right]+\;j_{\text{acatm}})\;}-k_{\text{deatm}}\left(1+[\text{Wip}1]\right)\frac{[\text{AT}M^{*}\;]\;\;}{\;([\text{AT}M^{*}]+j_{\text{deatm}})}$
3	$\frac{d\left[p53^{*}\right]}{dt}=k_{\text{acp}531}\frac{[\text{AT}M^{*}]\;}{\left([\text{AT}M^{*}]+j_{\text{atm}}\right)}\left[p53\right]-k_{\text{dep}53}\left[p53^{*}\right]-k_{\text{dp}53s}[\text{Mdm}2_{n}]\frac{\left[p53^{*}\right]}{j_{1p53n}+\left[p53^{*}\right]}$
4	$\frac{d\left[p53\right]}{dt}=k_{\text{sp}53}-k_{\text{dp}53n}\left[p53\right]-k_{\text{dp}53}[\text{Mdm}2_{n}]\frac{\left[p53\right]}{j_{1p53n}+\left[p53\right]}-k_{\text{acp}53}[p53]+k_{\text{dep}53}[p53^{*}]$
5	$\frac{d\left[\text{Wip}1\right]}{\text{dt}}=k_{\text{swip}10}+k_{\text{swip}1}\frac{{\left[p53\text{arrester}\right]}^{3}}{\;(j_{\text{swip}1}^{3}+{\left[p53\text{arrester}\right]}^{3})}\;-k_{\text{dpwip}1}\left[\text{Wip}1\right]$
6	$\frac{d\left[\text{Mdm}2_{n}\right]}{\text{dt}}=k_{i}\left[\text{Mdm}2_{\text{cp}}\right]-k_{0}\left[\text{Mdm}2_{n}\right]-k_{\text{dmdm}2n}\left[\text{Mdm}2_{n}\right]$
7	$\left[\text{ATM}\right]=\text{AT}M_{\text{tot}}-2\left[\text{AT}M_{2\;}\right]-[\text{AT}M^{*}]$
8	$k_{\text{dmdm}2n}=k_{\text{dmdm}2n0}\;+k_{\text{dmdm}2n1}\;\frac{\left[\text{ATM}{\;}^{*}\right]}{\left([\text{AT}M^{*}]+\;j_{\text{atm}}\right)}$
9	$\left[p53^{*}]\;\;=\;[p53\text{arrester}]\;+\;[p53\text{killer}\right]$

**Table 2:** Three modes of core oscillator subsystem given in Table 1

	p53* Dynamics	The value of $n_{c}$	Settings for (9) in Table 1
1	low steady state of [p53*]	0	[p53*] = [p53arrester] with [p53killer] = 0
2	oscillations of [p53*]	20	[p53*] = [p53arrester] with [p53killer] = 0
3	high steady state of [p53*]	20	[p53*] = [p53killer] with [p53arrester] = 0

The two‐phase dynamics of p53 network might be thought as a strategy giving cell a time to recover from the stress before the decision for apoptosis [7]. In two‐phase dynamics model, if the damage is repaired in a certain amount of time, then the p53 level goes back to its low basal level; otherwise, the cell goes to the second phase driving the concentration of p53 to a (sustained) high level, which is required for apoptosis. As demonstrated by the numerical solution of active p53 concentration, i.e. [p53*], in Fig. 1*b*, the Zhang's model, which is given in the supplementary material of [6], describes well the mentioned two‐phase dynamics of p53 network. The digital pulses shown in Fig. 1*b* are obtained by numerically solving the 17D Zhang's model by disregarding the repair subsystem and setting the number $n_{c}$ (the number of DSBCs) to 20. Herein, the ordinary differential equations defining the models are solved by ode45 solver available in MATLAB^®^ numerical software environment.

In the block diagram representation of Zhang's model in Fig. 1*a*, each subsystem represents a set of differential equations and the direction of arrow shows the transfer of a variable from one subsystem to the other. To review Zhang's model in a concise way, we divide the model into functionally meaningful subsystems: (i) repair subsystem, (ii) ATM subsystem, (iii) p53 subsystem, (iv) Wip1 subsystem, (v) P53DINP1 subsystem, (vi) PTEN subsystem, (vii) Mdm2 subsystem, (viii) p21 subsystem and (ix) caspase subsystem. Three feedback loops are identified: (i) ATM‐p53‐Wip1 feedback loop which is called shortly as Wip1 feedback loop with the name of feedback variable and constituted by ATM sensor subsystem, p53 subsystem and Wip1 subsystem, (ii) p53‐PTEN‐Akt‐Mdm2 feedback loop which is called as PTEN feedback loop as constituted by P53DINP1 subsystem, PTEN subsystem and Mdm2 subsystem and (iii) the feedback loop between p53 subsystem and Mdm2 subsystem which is called as p53‐Mdm2 feedback loop.

The rest of Section 2 is devoted to the recognition of the core oscillator subsystem of 6D underpinning the oscillatory dynamics of Zhang's full model [6] by segregation of the subsystems. It should be first noted that ATM‐p53‐Wip1 feedback loop is responsible for the oscillations in the first phase, while p53‐PTEN‐Mdm2 feedback loop becomes dominant in the second phase [6]. Since there is a sequential predominance of feedback loops, to analyse the oscillations in the first phase, PTEN feedback loop can be ignored for the sake of simplicity [6]. Focusing on the first phase, core oscillator subsystem of p53 network can be extracted by isolating ATM‐p53‐Wip1 feedback loop from the p53‐PTEN‐Akt‐Mdm2 feedback loop. To do so, the state variables p53killer and Mdm2_n_ that are transferred from ATM‐p53‐Wip1 feedback loop are held constant (see Fig. 1*c*). We set [Mdm2_n_] as constant at its prescribed initial value of 0.26 and [p53killer] at zero. It is observed from our simulations that the small deviations in these two parameters do not change the qualitative behaviour of the first phase dynamics. It can be seen from Fig. 1*a* that p21 and caspase subsystem do not provide any feedback signal to the p53 network, having no effect on p53 dynamics, so can be ignored too, as in Fig. 1*c*. Table 1 presents the resulting isolated model that is the core oscillator subsystem of the Zhang's full model. As shown in Fig. 1*d*, the obtained 6D oscillator subsystem is able to show all of the three modes, namely the equilibrium at the low level of [p53*], the oscillation and the equilibrium at the high level of [p53*], depending on the values of $n_{c}$, [p53killer] and [p53arrester] in P53DINP1 subsystem, as indicated in Table 2. It should be noted that, in the Zhang's full model, the value of $\;n_{c}$, p53arrester and p53killer are controlled by other subsystems.

## 3 Dimensionality reduction

In 17D two‐phase model by Zhang *et al.* [6], different modes of p53 dynamics result in different outcomes: oscillation of p53arrester level results in cell cycle arrest via stimulating p21 [3, 8], high steady state of p53killer level results in apoptosis via stimulating caspase mechanism [9–11] and low level of p53* indicates normal cell cycle progression [12, 13]. These consequences of the two‐phase dynamics can be correlated to the p53* levels and a few other variables such as ATM and Wip1 only, rather than a variety of highly possible superfluous variables by getting rid of downstream elements [5]. One of the main aims of this paper is to introduce a 2D model that solely describes these modes of p53 dynamics in a compact and efficient way ignoring non‐essential components. The idea behind this observation of the possibility of reducing the Zhang's model defined with 17 differential equations into a 2D model relies on the fact that any continuous time integer‐dimensional dynamical system is in some sense, qualitatively equivalent to an oscillatory 2D dynamical system.

### 3.1 Reduction to 2D model

**Fig. 2:**
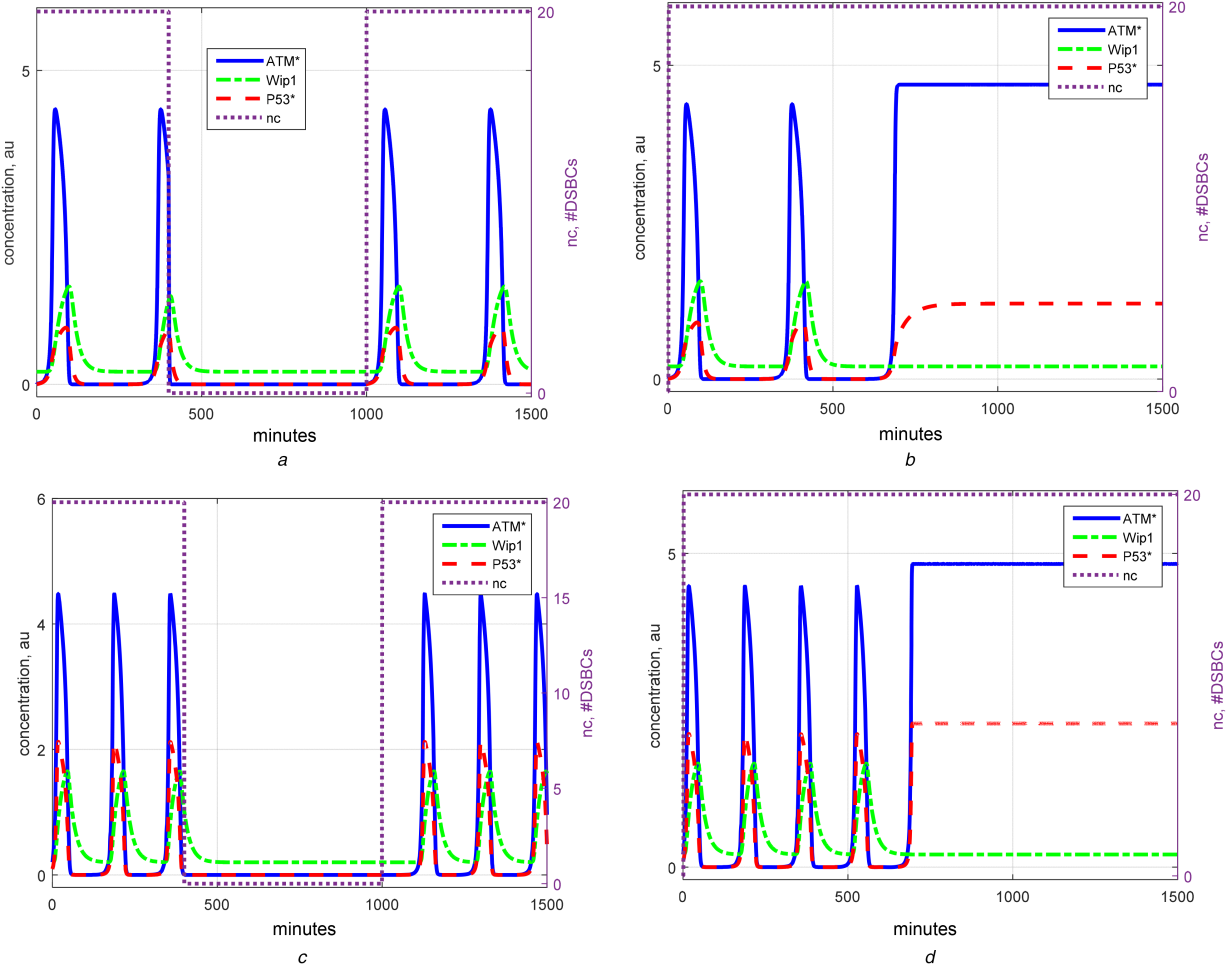
Time evolutions of [p53*], [ATM*] and [Wip1] in the 2D reduced ATM–Wip1 oscillator model. Setting in (a) and (c): $n_{c}=20$ *for 0 <t <400 and 1000 < t < 1500, and* $n_{c}=0$ *for 400 < t < 1000, where t stands for time in minutes. The model shows or ceases oscillation at proper* $n_{c}$ values. [p53*] Graph indicates also time evaluation of [p53arrester] since [p53killer] is zero and so [p53*] = [p53arrester]. Setting in (b) and (d): $n_{c}=20$ *for all time and* [P53arrester]=0 *for t > 600, apoptosis is initiated at t = 600. For t < 400,* [P53∗]=[P53arrester] *but for t > 600,* $\left[P53^{*}]\;=\;[P53killer\right]$ *according to the constraint of (10) in Table* 2 ***(a)*** 6D oscillator subsystem showing oscillations as well as recovery of low level [p53*] mode after DNA damage is repaired, ***(b)*** 6D oscillator subsystem showing two‐phase dynamics, ***(c)*** 2D reduced oscillator model showing oscillations as well as recovery of low level [p53*] mode after DNA damage is repaired, ***(d)*** 2D reduced oscillator model showing two‐phase dynamics

Reduction of high‐order systems into low‐order ones is done to simplify complex systems for the purpose of easy analysis and understanding essential components of the systems which may be helpful in further cancer research. In a successful reduction, the simplified system displays the same qualitative behaviour with a smaller number of state variables and parameters. This section introduces a reduced 2D differential equations model of ATM and Wip1 variables, which possesses the same qualitative behaviour with that of 6D isolated oscillator subsystem described in Section 2. The obtained 2D model is given in Table 3 and details of the reduction process are given in the supplementary material. In our 2D model, ATM directly signals to Wip1 via algebraic [p53*] equation [i.e. (3) of Table 3]. With appropriate settings of parameters given in Table 2, this simple model of ATM and Wip1 successfully elucidates the three modes of p53 dynamics, namely the modes of low [p53*] level, [p53*] oscillations and high level of [p53*]. See Figs. 2*c* and *d* for the time evolutions of [p53*], [ATM*] (i.e. phosphorylated ATM) and [Wip1] in the three modes of the introduced 2D model, and Figs. 2*a* and *b* for those of the 6D model. The reduced 2D model successfully inherits the qualitative behaviour of the two‐phase dynamics of the Zhang's model with an expense of the changes in the amplitude and frequency of the signals. It should be noted that the original amplitude and frequency might be reconstructed by scaling the time and range of the variables.

**Table 3:** Introduced 2D oscillator model of two‐phase p53 dynamics

Equation number	Equations
1	$\begin{array}{rl} \frac{d\left[\text{AT}M^{*}\right]}{dt} & =k_{\text{acatm}}\;\;\frac{n_{c}}{\left(n_{c}\;+\;jn_{c}\right)}[\text{AT}M^{*}]\;\frac{\sqrt{0.1*\left(\text{AT}M_{\text{tot}}-\left[\text{AT}M^{*}\right]\right)}}{\left(\sqrt{0.1*\left(\text{AT}M_{\text{tot}}-\left[\text{AT}M^{*}\right]\right)}+\text{jacatm}\right)}\;\\ & \;-k_{\text{deatm}}\;\left(\;1\;+[\;\text{Wip}1]\;\right)\frac{\left[\text{AT}M^{*}\right]}{\left([\text{AT}M^{*}]\;+\;j_{\text{deatm}}\right)} \end{array}$
2	$\frac{d\left[\text{Wip}1\right]}{dt}=k_{\text{swip}10}+k_{\text{swip}1}\frac{{\left[p53\text{arrester}\right]}^{3}}{\left({\;j}_{\text{swip}1}^{3}+{\left[p53\text{arrester}\right]}^{3}\right)}-k_{\text{dwip}1}\left[\text{Wip}1\right]$
3	$\left[p53^{*}]\;=\left[p53\text{arrester}\right]+\left[p53\text{killer}\right]=[\text{AT}M^{*}](1.5-3.947[\;\text{Mdm}2_{n}\;]\right)$

According to Harris and Levine [14], there are at least seven negative feedback loops and three positive feedback loops. Among these feedback loops, p53‐Mdm2 negative feedback loop, Wip1 negative feedback loop and PTEN positive feedback loop are the ones that are included and associated with specific distinguishable functionalities in two‐phase dynamics model by Zhang *et al.* [6].

The combination of negative and positive feedbacks is known to produce robust oscillations [15]. For example, PTEN feedback loop makes the apoptosis more robust by increasing p53 levels in the second phase [6]. In other p53 network models, also the effect of added feedback loops is studied. For example, Kim and Jackson [16] show that p53 oscillations become more robust when a positive feedback loop (Ror‐alfa) is added. Also, Zhang e*t al.* [17] demonstrates that additional positive feedback loops in different models increase the robustness of the oscillations. In the case of the introduced 2D oscillator based on two‐phase dynamics, two feedback loops are responsible for oscillations: (i) bistability of ATM, which includes a positive feedback loop intrinsic in its dynamics and (ii) negative feedback loop of Wip1. In addition to the oscillatory dynamics, we showed that this minimal model structure alone is capable of generating other qualitative p53 dynamics as well such as low and high equilibriums (see Fig. 2). The proposed simplification of the Zhang's model down to the network of ATM and Wip1 variables is in agreement with the experimental work by Shreeram *et al.* [13], in which they show that the mutual relationship between ATM and Wip1 plays an important role in tumorigenesis. In this regard, we think that the ATM–Wip1 interaction provides the flexibility of qualitative p53 dynamics while other loops make this structure more robust.

In the 2D model, we characterise [p53*] as with an algebraic equation in a way that it has few but operationally well‐defined parameters (see (3) of Table 3). While [p53*] is inversely correlated with [Mdm2_n_], it is directly proportional to [ATM*]. Therefore, the ATM dynamics reflects on [p53*]. [p53*] oscillates whenever [ATM*] oscillates. When [ATM*] is in high or low steady state, [p53*] is at high or low level, respectively. This is also consistent with the experimental observation that ATM is the main upstream signals of p53 and p53 pulses originate from recurrent initiation of ATM [3].

### 3.2 Phase space analysis of the 2D reduced oscillator model: characterisation of relaxation oscillation

In this section, first bistable dynamics of [ATM*] is analysed by employing dynamic route approach to (1) of Table 3 by quasi‐steady‐state assumption for [Wip1] variable and then oscillatory ATM*–Wip1 interaction is demonstrated by means of 2D phase portraits.

**Fig. 3:**
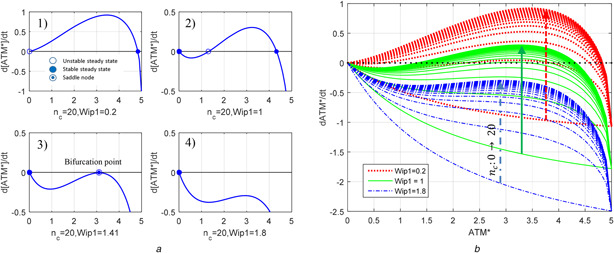
Dynamic routes of [ATM*] under different values of Wip1 and $n_{c}$ *describing the mechanism of ATM–Wip1 oscillations* ***(a)(a.1)*** One unstable, one asymptotically stable equilibria in the non‐negative region of [ATM*] when $n_{c}=20$ and Wip1=0.2, ***(a.2)*** One unstable, two asymptotically stable equilibria in the non‐negative region of [ATM*] when $n_{c}=20$ and Wip1=1, ***(a.3)*** One asymptotically stable, a double saddle equilibria in the non‐negative region of [ATM*] when $n_{c}=20$ and Wip1=1.41, ***(a.4)*** One asymptotically stable equilibrium in the non‐negative region of [ATM*] when $n_{c}=20$ and Wip1=1.8, ***(b)*** Three sets of dynamic routes of [ATM*]; first one obtained for Wip1=0.2 and $n_{c}\in [0,20]$, the second for Wip1=1 and $n_{c}\in [0,20]$ and the third for Wip1=1.8 and $n_{c}\in [0,20]$

As depicted in Fig. 3, [ATM*] dynamics has one or two stable equilibrium points in a physiologically meaningful range (i.e. the set of non‐negative [ATM*] values) depending on the values of $n_{c}$ and [Wip1]. Characteristics of [ATM*] dynamics change as with the combined effect of Wip1 and $n_{c}$ (see Fig. 3*b*). As $n_{c}$ phosphorylates ATM and drives its concentration to a high steady state; Wip1 dephosphorylates ATM* and drives its concentration to low steady state even in the presence of DNA damage (i.e. the case of high $n_{c}$ value) [13] (see Fig. 3*b*).

Dynamic route approach applied to [ATM*] dynamics for typical Wip1 values leading different sets of equilibrium dynamics for [ATM*] can assist to show how oscillations are produced (see Fig. 3*a*). Basal level of Wip1 is chosen as 0.2 and $n_{c}$ is set to 0 for representing the situation before the damage. In this initial condition, [ATM*] has only one equilibrium point at zero which is asymptotically stable (see the bottom curve of the upper red dotted bunch of [ATM*] graphs in Fig. 3*b*). When setting $n_{c}$ to 20 to represent the damage, [ATM*] characteristics change as: equilibrium at zero becomes unstable and, under arbitrary small perturbation, [ATM*] goes up to the asymptotically stable equilibrium at the high steady‐state value (Fig. 3*a*.1). As [ATM*] goes into this high steady state, at the same time, in accordance to (2) and (3) of Table 3, [Wip1] increases. So, it pulls the curve of [ATM*] rate (i.e. $d[\text{AT}M^{*}]/dt$) downward first creating a saddle point at an intermediate [ATM*] value (see Fig. 3*a*.3) and then leading the zero equilibrium point of [ATM*] to become asymptotically stable (see Fig. 3*a*.4). Zero value of [ATM*] relaxes [Wip1] to its rest state of 0.2, and this ATM*–Wip1 interaction repeats itself over again causing oscillations.

**Fig. 4:**
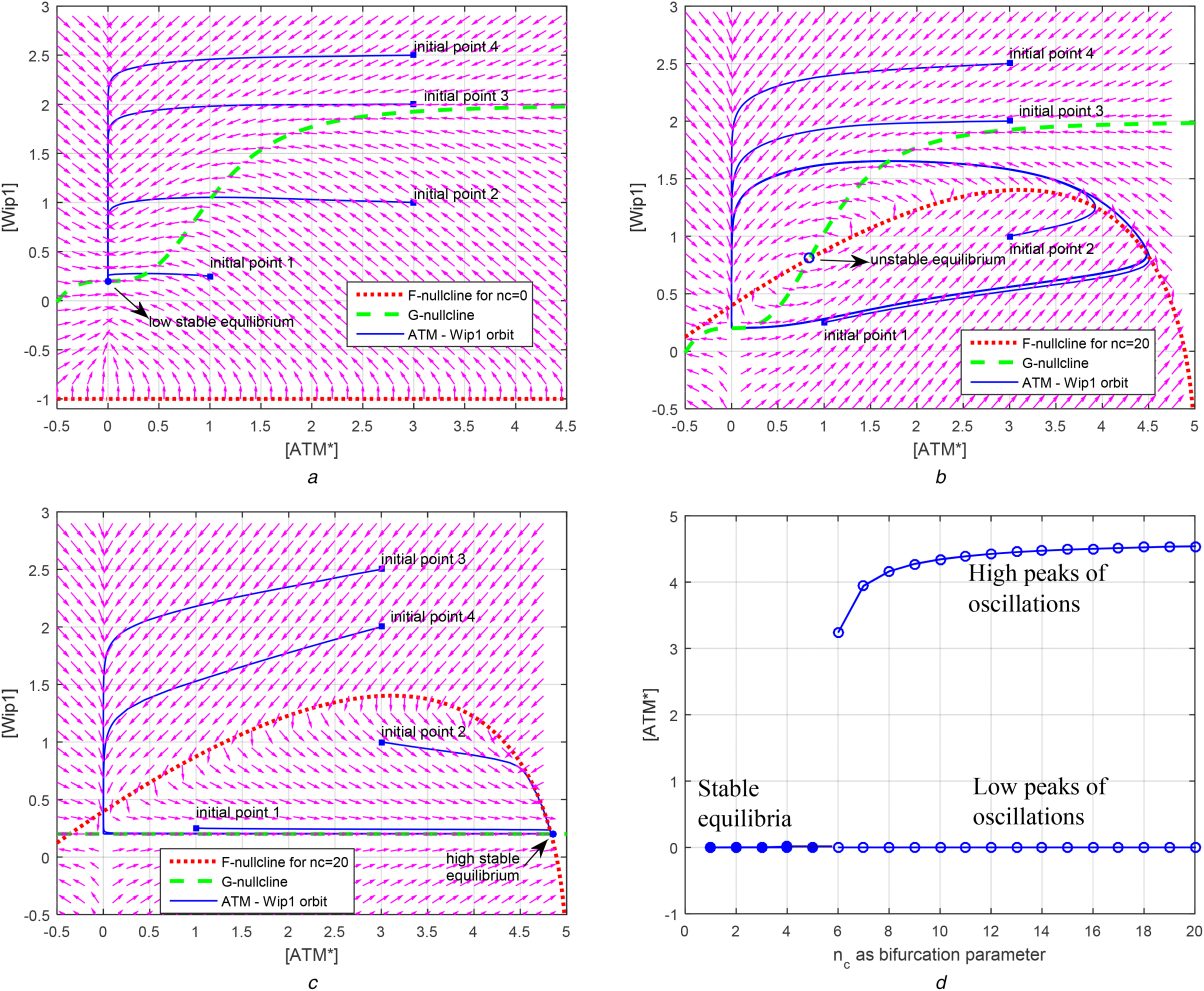
Bifurcation of dynamics of relaxation oscillator based on the positions of F‐, G‐ and [ATM*] = 0 nullclines. Arrows in the phase portraits of (a)–(c) represent the vector field. Note that initial point of [ATM*] cannot exist ATM_tot_, which is chosen to be 5 by Zhang et al. [1], *since the term* $\left(\text{AT}M_{\text{tot}}-\left[\text{AT}M^{*}\right]\right)$ *in* 1 *of Table* 3 *is under square root* ***(a)*** When $n_{c}$ = 0, F‐ and G‐nullclines do not intersect in the (physically meaningful) first quadrant. In this situation, there is a unique stable equilibrium in the first quadrant located at [Wip1] = 0.2 and [ATM*] = 0, ***(b)*** When $n_{c}$ is increased enough, F‐nullcline shifts upward and intersects with G‐nullcline in the first quadrant at the unstable equilibrium. For $n_{c}$ = 20, eigenvalues of the Jacobian of the model in Table 3 at this equilibrium are 0.0122−j0.1040 and 0.0122+j0.1040, indicating that the equilibrium is unstable, so repelling the trajectories in the first quadrant toward the limit cycle, ***(c)*** In case of [p53arrester] = 0 and $n_{c}=0$, G‐nullcline shifts downward until it intersects F‐nullcline at a stable equilibrium ([ATM*], [Wip1])≅(4.9, 0.2). The eigenvalues of the Jacobian at this point are −3.2935 and −0.0500 indicating that equilibrium is stable, attracting all trajectories in the first quadrant, ***(d)*** All‐or‐none dependence of the amplitude of [ATM*] oscillation on the strength of external stimulus $n_{c}$

In the phase space, when external stimulus $n_{c}$ is 20, nullclines of 2D oscillator equations given in Table 4 intersect at unstable fixed point (see Fig. 4*b*). Stable limit cycle around this unstable fixed point is seen to appear according to the Poincare–Bendixson theorem by considering a closed‐bounded region enclosing without including the fixed point (see Fig. 4*b*). Note that Poincare–Bendixson theorem is stated as ‘a 2D system exhibits limit cycles if it is confined in a closed‐bounded region that does not contain any fixed point’.

**Table 4:** Nullclines of 2D oscillator model

Equation number	Equations
1	F‐nullcline (with $\mu_{1}\;=\;\frac{k_{\text{deatm}}}{\;\left(\left[\text{AT}M^{*}\right]+j_{\text{deatm}}\right)}$):
	$F\left(\left[ATM^{*}\right],\;n_{c}\right)-[Wip1]=\frac{1}{\mu_{1}}$ $\left(k_{\text{acatm}}\;\frac{n_{c}}{\left(n_{c}+jn_{c}\right)}\;\frac{\sqrt{0.1*(\text{AT}M_{\text{tot}}-\left[\text{AT}M^{*}\right])}}{\left(\sqrt{0.1*(\text{AT}M_{\text{tot}}-\left[\text{AT}M^{*}\right])}\;\;\;\;+\;\text{jacatm}\right)}\;-\mu_{1}\right)-[\;\text{Wip}1]=0$
2	[ATM*] = 0
3	G‐nullcline (with $\mu_{2}=k_{\text{dwip}1}$):
	$G\left(\left[p53\text{arrester}\right],\;\left[\text{AT}M^{*}\right]\right)-[\;\text{Wip}1]=$ $\frac{1}{\mu_{2}}\left(k_{\text{swip}10}+k_{\text{swip}1}\frac{{\left[p53\text{arrester}\right]}^{3}}{\;(j_{\text{swip}1}^{3}+{\left[p53\text{arrester}\right]}^{3})}\;\right)-[\;\text{Wip}1]=0$

**Fig. 5:**
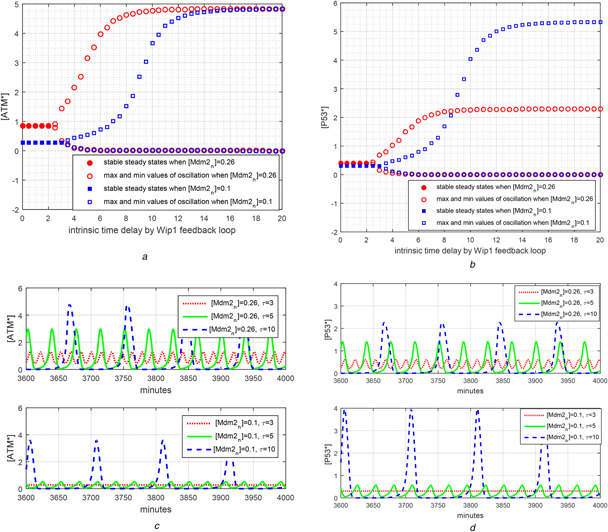
Changes in amplitudes and frequencies of [ATM*] and [p53*] oscillations depending on time delay and [Mdm2_n_] ***(a)*** Oscillation starts at *τ* = 2 and 3 for [Mdm2_n_] = 0.26 and 0.1, respectively. As time delay of Wip1 feedback loop increases, the amplitude of oscillations increases, eventually saturating at the high stable equilibrium state of [ATM*] dynamics given by (1) of Table 3. Low Mdm2_n_ level of 0.1 in the first phase has a potential to decrease amplitudes of oscillations in case of minor delay of Wip1 feedback loop. Therefore, enhanced level of [Mdm2_n_] is good for having high amplitude oscillations for [ATM*], ***(b)*** Low level of [Mdm2_n_] increases the amplitudes of [p53*] oscillations, so leading anti‐tumour activity, only when the time delay provided by Wip1 exceeds 8 min, ***(c)*** Time evaluation for [ATM*] is sketched to see the frequency information. 1D SDD equation in Table 5 is solved until 4000 min long to give time for transient effects to disappear. 1D SDD model exhibits oscillation at *τ* = 3 min for [Mdm2_n_] = 0.26 but not for [Mdm2_n_] = 0.1. A larger amplitude of oscillations for [Mdm2_n_] = 0.26 is obtained at *τ* = 5 min compared with [Mdm2_n_] = 0.1. At *τ* = 10 min, the amplitude of [ATM*] oscillation reaches its maximum value. Since *τ* is relatively high, at both the values of [Mdm2_n_], ATM dynamics can have enough time to reach its high equilibrium state. As *τ* increases, the period of oscillations increases too. Also, [Mdm2_n_] has similar effect for some *τ* values. At *τ* = 5 min, the period is about 28 min for [Mdm2_n_] = 0.1, the period is around 36 min for [Mdm2_n_] = 0.26, ***(d)*** [p53*] oscillations are weak when [Mdm2_n_] = 0.1 and *τ* is 5 min. However, when *τ* is 10 min, i.e. a large value, amplitudes of [p53*] oscillations are enhanced for both [Mdm2_n_] values

Since Wip1 whose dynamics is defined in (2) of Table 3 has a slower dynamics providing a proper intrinsic time delay, [ATM*] is able to switch between the above‐mentioned high and low steady states thus forming repeated pulses. Therefore, p53 network model owes its oscillations to the interaction between the bistable characteristic of fast ATM dynamics and intrinsic time delay of slow Wip1 negative feedback loop. This topological structure of ATM*–Wip1 resembles a bistable frustrated unit model that is able to exhibit relaxation oscillations [18]. Being a frustrated unit, one can freely choose or disturb model parameters provided that this does not violate the existence of two main sources of oscillations, namely the bistability of [ATM] and a proper time delay in Wip1 feedback loop. A sufficient amount of relaxation time (i.e. the time delay) is necessary for [ATM*] to reach the high steady state on every pulse. If the relaxation time is not sufficiently long, [ATM*] will switch back to zero value before reaching the high steady state, resulting in oscillations of smaller amplitudes (see Fig. 5). That is, the relaxation time determines the strength of the oscillations in the identified relaxation oscillator.

**Table 5:** 1D SDD differential equation counterpart of 2D oscillator model

Equation number	Equations
1	$\frac{d\left[\text{AT}M^{*}\right]}{dt}=\mu_{1}\left(F\left(\left[\text{AT}M^{*}\right],\;n_{c}\right)-\left[\text{Wip}1\right]\right)[\text{AT}M^{*}]$
2	$\left[p53\text{arrester}](t)\;=\;[\text{AT}M^{*}](t-\tau )(1.5-3.947[\text{Mdm}2_{n}]\right)$
3	$\;\left[\text{Wip}1\right]=\;\frac{1}{k_{\text{dwip}1}}\left(k_{\text{swip}10}+k_{\text{swip}1}\frac{{\left[p53\text{arrester}\right]}^{3}}{\;({j\;}_{\text{swip}1}^{3}+{\left[p53\text{arrester}\right]}^{3})}\right)\;$

Since the oscillatory response happens due to the transition from stable (Fig. 4*a*) to unstable (Fig. 4*b*) equilibrium located at the intersection point of the two nullclines, the system gives a complete oscillatory response or no response at all and the external stimulus, $n_{c}$, does not appreciably influence the amplitude of this relaxation oscillation (Fig. 4*d*). This is known as all‐or‐none phenomenon that is biologically an intriguing property of relaxation oscillators [19, 20]. In fact, the observations in wet‐lab experiments support the existence of a relaxation oscillator in p53 network. For example, Lahav *et al.* [2] states that ‘The fixed mean height and duration of p53 pulses do not depend on the amount of DNA damage’ and Geva‐Zatorsky *et al.* [21] state that ‘The peak amplitude and timing did not depend on the dose of irradiation’. The underlying mechanism for such observation is strong amplification/saturation mechanism of the ATM module that gives signal of almost the same strength to p53 module regardless of the DSB numbers [22].

The introduced 2D oscillator model is able to exhibit not only the digital pulses but also low and high equilibrium states of [p53*] which are required for normal cell growth and for apoptosis, respectively. When [p53arrester] = 0 (or close to zero), Wip1 feedback loop shuts off, the model dynamics is governed only by [ATM*] equation that has bistable characteristics. In this case, the value of $n_{c}$ determines whether [ATM*] will have a high or low stable equilibrium steady‐state value. If $n_{c}$ = 20 when Wip1 feedback loop shuts off, F‐ and G‐nullclines intersect at the high stable equilibrium state of [ATM*] (Fig. 4*c*). [ATM*] goes to high equilibrium state, so does [p53*] in accordance to the algebraic relation in (3) of Table 3. Since p53arrester is forced to be zero, all [p53*] become [p53killer] ready to trigger apoptosis.

One of the characteristics of the relaxation oscillators is that the relaxation time determines the frequency and amplitude of the oscillation. In the proposed ATM*–Wip1 oscillator, the variable [Wip1] and the constant parameter [Mdm2_n_] both contribute to the relaxation time. [Mdm2_n_] contributes to the relaxation time via Wip1 equation [(2) of Table 3]. To investigate separately the contributions of these two determinants to the relaxation time and oscillations, we reduce the 2D ATM*–Wip1 oscillator model into the 1D state‐dependent delay (SDD) differential equation of [ATM*] in Table 5 by explicitly indicating the time delay contribution *τ* of Wip1 and keeping [Mdm2_n_] as a parameter (See [23] for general SDD differential equations.). It should be noted that the intrinsic time delay of Wip1 feedback loop models the relaxation time of the slow dynamics part of the relaxation oscillator. Taking *τ* as a bifurcation parameter for different [Mdm2_n_] values, Figs. 5*a* and *c* show that as [Mdm2_n_] increases, the amplitude of [ATM*] oscillations continues to increase and saturates at large values of *τ*. However, the effect of [Mdm2_n_] on [p53*] oscillations is rather complex. Depending on the value of *τ*, [Mdm2_n_] enhances or weakens [p53*] oscillations (Figs. 5*b* and *d*). For *τ* between 3 and 8 min, [p53*] oscillations are enhanced by [Mdm2_n_]; however, for *τ* >8 min the oscillations are weakened by [Mdm2_n_]. For the parameters of the reduced ATM*–Wip1 oscillator equations in Table 3, the intrinsic time delay of Wip1 is around 4 min, so abundance of [Mdm2_n_] increases the amplitudes of [p53*] oscillations. Although [Mdm2_n_] is a negative regulator of p53, it has a positive effect on [p53] oscillations thus on cell cycle arrest. Then, we conclude that Mdm2 is good for enhancing DNA‐damage response, and hence considered as a tumour suppressor. These results correlate with the biological findings. In wet‐lab experiments, it has been observed that Mdm2 may exert effects that suppress cell proliferation [24] and mitotic progression as well [24–26]. In addition, Manfredi [24] showed that loss of Mdm2 enhanced tumour formation, which may result from a weak cell cycle arrest signal.

## 4 Determinants of two‐phase dynamics of p53 network

Zhang *et al.* [6] argues that the first and second phase of the two‐phase dynamics of 17D p53 network model becomes active depending on the relative strengths of Wip1 and PTEN feedback loops. In contrast to the study by Zhang *et al.* [6], Section 4 of this paper describes the phases based on a 2D oscillator model excited by DSBCs as its two different qualitative modes in the following way. (i) Wip1 feedback loop, which feedbacks ATM to itself, is the source of oscillation, which appears in the first phase, as also explained in [6]. (ii) The second phase appears after the oscillation stops due to the extinction of p53arrester by the accumulated activity of P53DINP1 no matter what the relative strength of PTEN feedback loop over Wip1 feedback loop is, opposing to the argument by Zhang *et al.* [6]. (iii) The role of PTEN feedback loop is two‐fold: the activation of PTEN feedback loop in the first phase decreases the amplitudes of the oscillation but not stop the oscillation while, in the second phase, it boosts up the level of equilibrium state of p53 to initiate apoptosis.

### 4.1 P53DINP1 acts as an OATGS

**Fig. 6:**
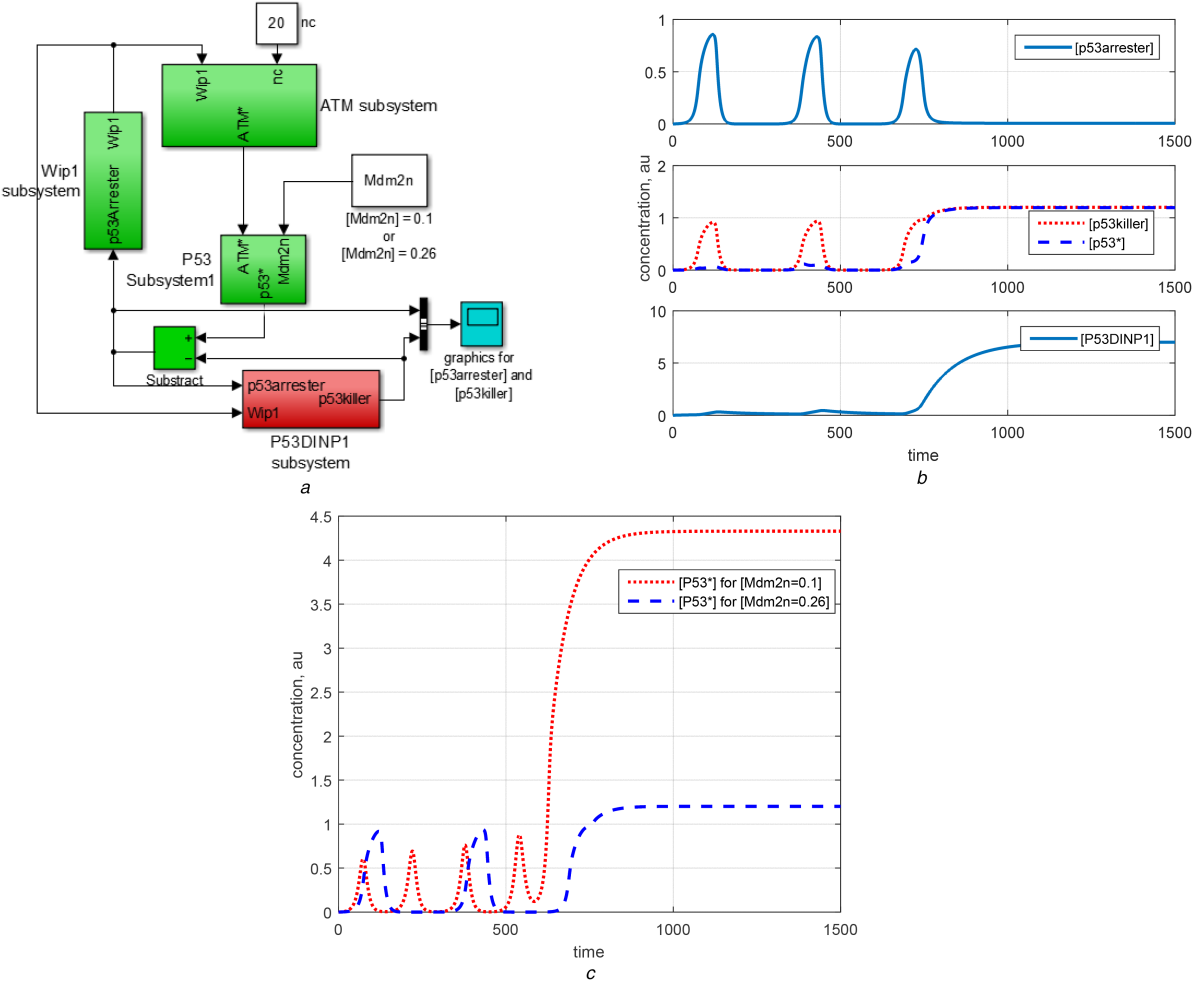
Core oscillator subsystem extended with the addition of P53DINP1 subsystem ***(a)*** Block diagram representation of core oscillator subsystem with P53DINP1 subsystem, ***(b)*** When included in the core oscillator subsystem, P53DINP1 activity automatically switches oscillator from oscillatory regime to the high steady state at around time, *t* = 600 min, due to the extinction of p53arresters. After the switching action, [p53*] value is greatly affected by [Mdm2_n_] value which is set by PTEN feedback loop, ***(c)*** Low [Mdm2_n_] value of 0.1 decreases the amplitude of oscillations but increases the [p53*] (i.e. p53killer) in apoptosis. When [Mdm2_n_] is 0.26, amplitude of oscillations is bigger but the [p53*] level in apoptosis is smaller

Since apoptosis is a decision, there must be a switching action, which requires the existence of a switch. Here, we reveal functional importance of P53DINP1 as a switch, more specifically an OATGS [27] in two‐phase dynamics. To demonstrate the switching action of P53DINP1, we add the dynamics of P53DINP1 and p53killer to the core oscillator subsystem defined in Table 1 (see Fig. 6*a*). The simulation results given in Figs. 6*b* and *c* show the followings. P53DINP1 activity increases as it accumulates over oscillations. When its activity becomes so high that it is able to turn all p53arresters in the environment into p53killers, then Wip1 feedback loop shuts off to stop oscillation. If $n_{c}$ is still high (e.g. 15 < $n_{c}$ < 20), [ATM*] goes to high steady state elevating [p53*] to a high level, too. This analysis supports that a potential cancer therapeutic approach can be used to elevate [P53DINP1] in order to drive cell to apoptosis. Note that the apoptotic importance of P53DINP1 is stated in the experimental findings in [28] and is patented [29].

### 4.2 PTEN feedback loop

When P53DINP1 activity is included into core oscillator subsystem, effect of Mdm2 can be automatically seen in the first phase and the second phase of p53 dynamics as in Fig. 6*c*. Low [Mdm2_n_] value of 0.1 decreases the amplitude of oscillations but increases the [p53*] (i.e. p53killer) at apoptosis. When [Mdm2_n_] is 0.26, the amplitude of oscillations is bigger but [p53*] level in apoptosis is smaller. So, to maintain a strong oscillation in the first phase and higher sustained value at apoptosis, [Mdm2_n_] value must be degraded just after the oscillator stops by the extinction of p53arrester, which is maintained by P53DINP1 activity in 17D two‐phase model. This indicates that the ultimate function of PTEN feedback loop activated by P53DINP1 in the second phase is to drop [Mdm2_n_] value to a very low value in order to drive [p53*] level to a higher value than the peaks of [p53*] oscillation. Fine distinction between the peaks of [p53*] oscillations in the first phase and constant high level of [p53*] in the second phase guarantees a more reliable decision of cell fate.

When P53DINP1 stops the oscillator such that [p53*] level is in high state (see Figs. 6*b* and *c*), the level of p53killer is not sufficient to trigger caspase mechanism for initiating apoptosis [6]. Therefore, [Mdm2_n_] must be decreased in the second phase to elevate [p53killer] to the higher steady state. We emphasise that switching to apoptosis and maintaining a proper high level to trigger apoptosis are two different things. This finding is very important in pointing out that stopping the oscillator to trigger apoptosis and providing a sufficient [p53*] level after the oscillator stops requires two different actions both of which are necessary and important. Thus, stopping the oscillator is the first critical step in initiating apoptosis.

In the 17D model of Zhang *et al.* [6], decreasing of [Mdm2_n_] is accomplished by activation of PTEN feedback loop in the second phase. A clear distinction to be done here: the activation of PTEN feedback loop does not cause a switching action from the first phase to the second phase but it maintains a higher level of [p53*] by reducing [Mdm2_n_] in the second phase. In fact, the activation of PTEN feedback has a negative effect on the first phase by weakening the oscillations, due to the relaxation time effect of Mdm2. So, PTEN feedback must be activated just after the switching action is done by P53DINP1 in contrast to the claim of Zhang *et al.* [6], in which the level of PTEN determines whether p53 acts as a pulse generator or a switch in the second phase.

## 5 Analysis of mutations in P53 network via 2D oscillator model and revealing possible cancer therapy strategies

It is known that sensitive parameters that change bifurcation points usually correspond to high‐frequency oncogenic mutations in reality [30]. These sensitive parameters change the phase space of the system drastically. For the developed 2D oscillator model of p53 network, the intersection type of nullclines determines the characteristics of the phase space and any parameter that significantly changes the location of nullclines may correspond to an oncogenic mutation. In the sequel, we show that mutations such as Wip1 overexpression and ATM deficiency can be modelled by changing the corresponding parameters in the 2D oscillator system resulting in the changes of phase space. We also evaluate the prediction capability of 2D oscillator model in representing the outcomes of possible therapeutic intervention strategies.

### 5.1 Wip1 overexpression and Wip1 downregulation

**Fig. 7:**
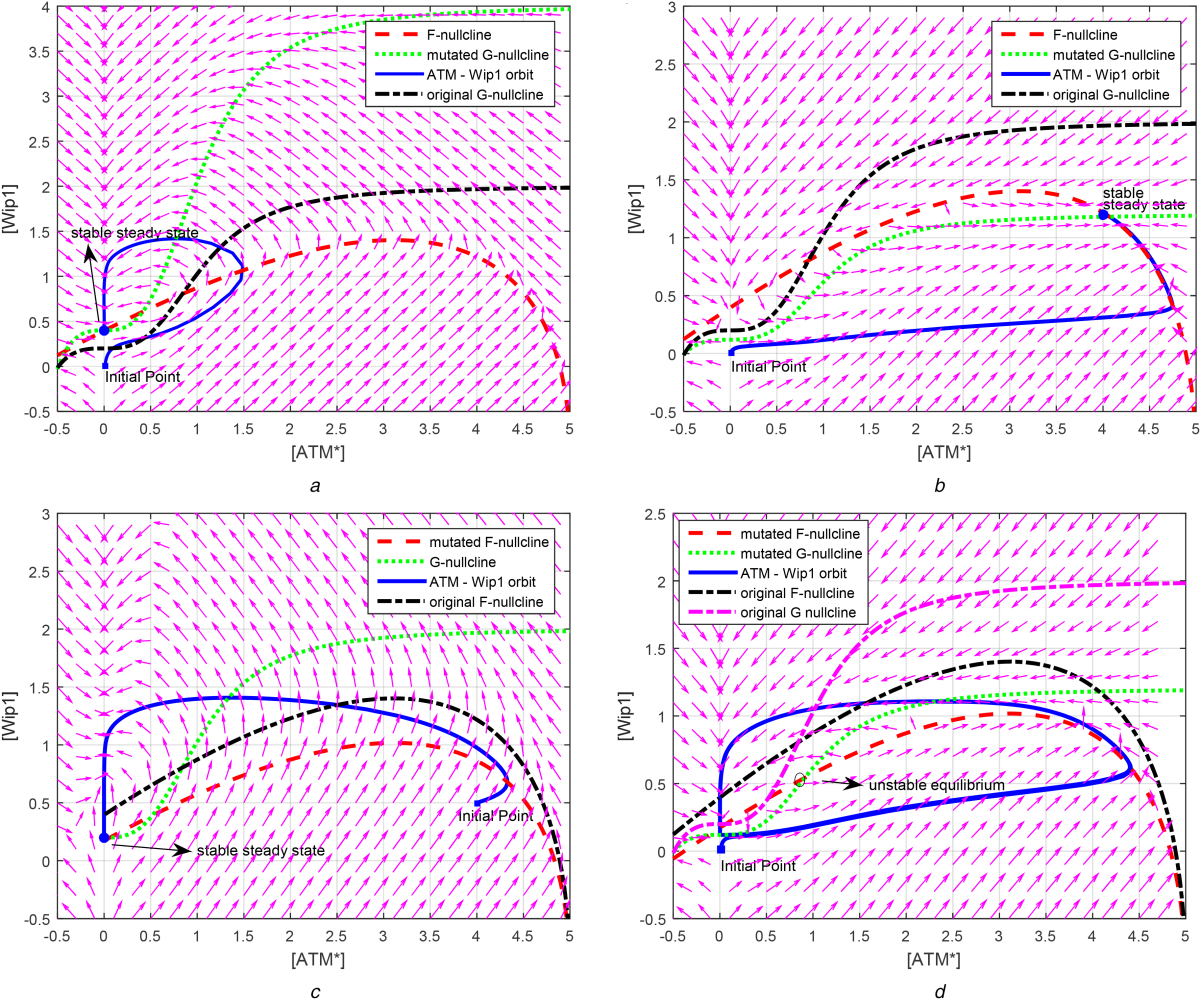
Effect of mutations to the phase space of 2D oscillator ***(a)*** Wip1 overexpression, *K*_wip1_ in (1) is 2, ***(b)*** Wip1 downregulation, *K*_wip1_ in (1) is 0.6, i.e. <1, ***(c)*** ATM deficiency, *K*_ATM_ in (2) is 5, ***(d)*** Degradation of Wip1 compensates ATM deficiency. Oscillatory response is recovered. *K*_wip1_ in (1) is 5 and *K*_ATM_ in (2) is 0.6

Wip1 overexpression is a mutation that is found in several types of cancers and has an oncogenic function [31–37]. The mutation is characterised by the high levels of Wip1 in cell. This situation can be embedded into our 2D model by increasing the Wip1 production rate via a constant *K*_wip1_ as shown in (1). Under DSBC activity, the increase in *K*_wip1_ moves the G‐nullcline upward, so changing the phase space of the system. Consequently, the system loses its ability to oscillate (see Fig. 7*a*). Since the p53 oscillations are important for arresting cell cycle [5], and the defects in cell cycle arrest are the prerequisite of cancer [33, 38, 39], we emphasise that Wip1 overexpression may cause cancer by removing the cell's ability to arrest cell cycle as also noted by Zhang *et al.* [6]. This analysis also supports the biological experiments by Bulavin *et al.* [37] and Xu and Baltimore [40] which confirm that Wip1 overexpression may cause tumorigenesis even if p53 gene of the cell functions normally. To recover the healthy phase space for p53 dynamics, G‐nullcline must be moved downward, which can be accomplished by increasing the degradation rate of Wip1 in (1). This observation is consistent with the experimental findings in [41] stating that Wip1 overexpression can be recovered by Wip1 degradation

(1) $$\frac{d\left[\text{Wip}1\right]}{dt}=K_{\text{Wip}1}*\left(k_{\text{swip}10}+k_{\text{swip}1}*\frac{{\left[p53\text{arrester}\right]}^{3}}{\left(j_{\text{swip}1}^{3}+{\left[p53\text{arrester}\right]}^{3}\right)}\right)\;-k_{\text{dwip}1}*\left[\text{Wip}1\right]$$

It is known that loss of Wip1 function makes the cells sensitive to DNA‐damage‐induced apoptosis [42, 43]. By decreasing Wip1 activity via decreasing *K*_wip1_ in (1), G‐nullcline moves downward and intersects F‐nullcline at a stable high steady state (see Fig. 7*b*). Since the oscillator stops at a high steady state, the cell can now trigger apoptosis. This analysis is also consistent with the findings that the depletion of Wip1 makes the cells sensitive to apoptosis [44]. The ability to go apoptosis easily makes the cells resistant to tumour formation [13, 45, 46]. Recently, via wet‐lab experiments, the work in [22] shows that silencing of Wip1 enhances the levels of proteins, e.g. ATM, in DNA‐damage response of the cell, emphasising the role of Wip1 in cell fate decision. The analysis here demonstrates in mathematical terms that Wip1 is an attractive chemotherapeutic target, agreeing with biological findings [47–51].

### 5.2. ATM deficiency

ATM deficiency is an ATM mutation, which is characterised by insensitiveness to the damage. This mutation can be embedded into our 2D model, by decreasing the sensitiveness of ATM to $n_{c}$_,_ via a constant *K*_ATM_ as in (2). The sensitiveness of ATM to $n_{c}$ can be decreased by increasing *K*_ATM_. As a result, F‐nullcline moves downward, so changing the phase space and removing the system's ability to oscillate and to arrest cell cycle (see Fig. 7*c*). This feature of the introduced 2D oscillator model is in agreement with the experimental results demonstrating that ATM mutations cause defective cell cycle checkpoint activation [38, 52, 53]

(2) $$\begin{array}{rl} \frac{d\left[\text{AT}M^{*}\right]}{dt} & =k_{\text{acatm}}\;\frac{n_{c}}{\left(n_{c}\;+K_{\text{ATM}}*jn_{c}\right)}[\text{AT}M^{*}]\;\frac{\sqrt{0.1*\left(\text{AT}M_{\text{tot}}-\left[\text{AT}M^{*}\right]\right)}}{\left(\sqrt{0.1*\left(\text{AT}M_{\text{tot}}-\left[\text{AT}M^{*}\right]\right)}+\text{jacatm}\right)}\\ & \;-\;\text{kdeatm}\;\left(\;1\;+[\text{Wip}1]\;\right)\frac{\left[\text{AT}M^{*}\right]}{\left([\text{AT}M^{*}]+j_{\text{deatm}}\right)} \end{array}$$

### 5.3 Degradation of Wip1 rescues ATM deficiency

Another prediction can be obtained from the phase space perspective: the moving of G‐nullcline downward can be compensated by moving F‐nullcline downward too, so resulting an unstable equilibrium again; thus, keeping the qualitative property of the phase space. To move F‐nullcline downward, Wip1 activity could be reduced by increasing *K*_wip1_ parameter value in the model of (1). As a result, the ability to oscillate is regained (see Fig. 7*d*). This feature of the introduced 2D oscillator model is consistent with the fact observed in the wet‐lab experiments of [54]: the absence of Wip1 rescues ATM deficiency phenotypes in mice. Although our 2D model is not detailed to account for all related proteins taking roles in the wet‐lab experiments, it is capable of pointing out in mathematical terms that the degradation of Wip1 can rescue ATM mutation, since the nullclines can be moved in appropriate directions to recover the phase space.

### 5.4 Effect of Mdm2 overexpression and downregulation on cell fate

**Fig. 8:**
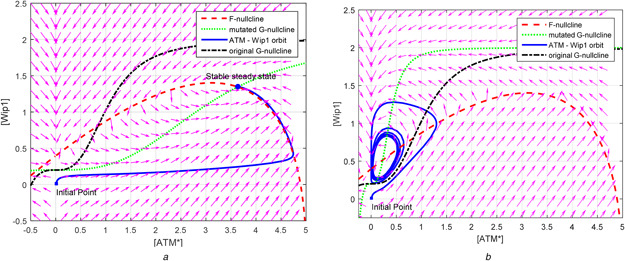
Overexpression and downregulation of Mdm2_n_ change the location of G‐nullcline and affect the phase space of oscillator ***(a)*** Oscillator stops at a high steady state when [Mdm2_n_] is overexpressed, e.g. [Mdm2_n_] = 0.34, in comparison with its normal values of 0.26, ***(b)*** Weaker oscillations of [ATM*] and [Wip1] are observed in case of downregulation of [Mdm2_n_], e.g. 0.05

Overexpression of Mdm2 is known to exist in some tumours [55]. Using bifurcation studies in a model without oscillatory dynamics, the work in [56] showed that Mdm2 overexpression keeps p53 level under the threshold levels of apoptosis or cell cycle arrest. Herein, we show the distorting effect of Mdm2 overexpression on oscillatory p53 dynamics. The effect of the overexpression of Mdm2 is modelled via the introduced 2D oscillator as: increase of [Mdm2_n_] stops the oscillations and drives the trajectories toward a relatively high steady state as shown in Fig. 8*a*. Since the oscillation stop due to a dysfunction of [Mdm2_n_], Mdm2_n_ cannot be utilised further in the second phase of the p53 dynamics to elevate [p53*] values to a higher level, resulting in a failed apoptosis. This result is in agreement with the study in [56], which reveals the mechanisms that make some cancer types with Mdm2 overexpression resistant to radiotherapy. In addition, this result emphasises the importance of PTEN feedback loop in avoiding overexpression of Mdm2 in the first phase by keeping Akt protein levels down [6].

In our 2D oscillator model, downregulation of Mdm2_n_ results in smaller amplitude oscillations (see Fig. 8*b*). On the other hand, stopping oscillations is the prerequisite of getting a high steady state of [p53*]. According to our model, decreasing p53 inhibitor (e.g. Mdm2_n_) levels does not cause a sustained high level of [p53*] for triggering apoptosis. This feature of 2D oscillator model implies that p53‐Mdm2 interaction, though it is structurally and biologically well understood [57], would affect p53 network in an unexpected way causing an additional level of complexity. Thus, we emphasise that Mdm2 does not have a straightforward suppressor–effector relation with p53 due to the relaxation oscillator nature of the 2D oscillator. This may be a source of confounding characteristics in p53 network that must be taken into account by experimentalists and it may be one of the reasons for why p53 network is becoming controversy, obsolete and more confusing as new experiments are done [58, 59].

### 5.5 Cancer therapy strategies that obey two‐phase dynamics

As we described previously, decreasing p53 inhibitors (e.g. Mdm2_n_) as a cancer therapeutic approach may have counter‐intuitive consequences hard to predict in the absence of a deep understanding of the p53 network. Without the knowledge of the current phase of the oscillator, degrading p53 inhibitors with the expectation of apoptosis may result in weak cell cycle arrest instead. To effectively use p53 inhibitors in therapies, it seems better to aim for apoptosis rather than just cell cycle arrest. For this purpose, a better drug to drive a cell to apoptosis would be a combination of drugs, which first stops the oscillator and then tries to degrade p53 inhibitors, suggesting that drug development process should take two‐phase dynamics into consideration cautiously. For this purpose, existing available methods that are able to target p53 inhibitors for degradation can be combined with the methods that target Wip1 feedback loop to shut off at the same time.

Also, as we showed in Section 5, some abnormalities that may cause cancer can be restored to a healthy state. In this regard, a drug that targets the oscillator to regain its healthy phase space would be invaluable. Such a drug development approach proposed in this paper requires a systems level understanding of p53 network, which employs computational studies.

It is known that a weak cell cycle arrest signal is the prerequisite of cancer [38]. So, strengthening the [p53*] oscillations may be an effective way of preventing cancer. A possible way to strengthen the amplitudes of oscillations may be to synchronise p53 network oscillator by another oscillator that has an effect on the amplitudes of [ATM*] oscillations. A candidate for having such an effect may be the circadian clock rhythm. Circadian clock and the DNA‐damage response are known to be connected through ATM [60] and it has been shown that cancer therapies work better if circadian rhythm is taken into account [61]. Since the circadian rhythm is produced by an oscillator and DNA‐damage response model involves an oscillator model (as shown in this paper), investigating the coupling of these two oscillators would be valuable and it may support the current wet‐lab experiments as well as making testable predictions for further experiments.

## 6 Discussion

Wip1, which is one of the oscillating variables in the proposed 2D model, is reported in the literature to exhibit non‐oscillatory dynamics in human osteosarcoma U2OS cell line after infrared (IR) exposure [22, 62]. On the other hand, oscillatory dynamics of Wip1 is observed on exposure of IR in some cell line studies [3, 62]. So, the model can be exploited as a theoretical framework for some particular cell lines that possess oscillatory Wip1 dynamics. It should also be noted that the models that have the ability to oscillate are likely to exhibit non‐oscillatory behaviours by tuning certain parameters. However, the models that lack the ability to oscillate need substantial changes to transform its dynamics into oscillations. So, considering the fact that both oscillatory and non‐oscillatory dynamics of Wip1 exist, oscillator models are more likely to also point out the mechanisms in non‐oscillatory dynamics of Wip1. The search for parameter space and mechanisms underlying the non‐oscillatory dynamics of Wip1 can be studied as future studies.

The study in [63] shows that if Wip1 synthesis rate is too high, oscillations are ceased. In agreement with [63], we showed in Fig. 5 that if Wip1 feedback loop is too fast (i.e. intrinsic time delay is too small), which can be the result of the increased synthesis rate of Wip1, the oscillations are ceased. Also, the studies done for Wip1 overexpression in Fig. 7*a* show that high synthesis rate of Wip1 causes oscillations to stop.

Wip1 feedback is shown to be indispensable for the oscillations; however, Mdm2 feedback alone is not sufficient to explain the oscillations, so Mdm2 feedback‐based oscillation models are questionable [3, 4]. We just isolate the system from Mdm2 dynamics to show a resulting complex dynamics implying that Wip1 is indispensable, whereas Mdm2 is dispensable for the existence of oscillations, as with proposing a model under the isolation of Mdm2 dynamics. However, it should be noted that there are models that rely on p53‐Mdm2 feedback loop and are also capable of showing qualitative behaviours of p53 network [64, 65].

## 7 Conclusion

In this paper, we proposed a reduced 2D oscillator model for p53 network that controls the cell fate. This reduced model is valuable for measuring the significance of the feedback loops, regulators and uncovering the essential working principles underlying the two‐phase dynamics. We show that, in two‐phase dynamics model, cell fate can be determined by modulating this 2D oscillator. We speculate that this oscillator constituting a core subsystem of p53 network may be considered as a potential cancer therapeutic target to control cell fate. By using the developed 2D oscillator model, we were able to post some cancer types as a phase space problem, which is a new kind of mechanistic explanation to those cancer types that result from deficient cell cycle arrest. We show that mutations in Wip1 and ATM drastically change the phase space of the oscillator. In addition, we reveal an interesting phenomenon about P53 inhibitors: the abundance of p53 inhibitors may increase the amplitudes of p53 oscillations. This may be one of the reasons for the complexity of p53 network. To qualitatively manipulate the p53 network, e.g. to drive the cell to apoptosis, one has to manipulate this core oscillator as a first step. Any other intervention strategies that do not address the working of the oscillator may give controversial results. Further research employing similar system level approaches on p53 network may lead to the development of novel therapeutic strategies. Another potential significance of the introduced simple 2D oscillator model for p53 network is the possibility of modelling and studying the couplings between p53 network and other oscillators such as circadian network.
